# Meeting the 24-h movement guidelines and health-related outcomes among youth with autism spectrum disorder: a seven-country observational study

**DOI:** 10.1186/s13034-022-00488-5

**Published:** 2022-06-23

**Authors:** Chunxiao Li, Justin A. Haegele, Fenghua Sun, Maria Luiza Tanure Alves, Stefanie Hwee Chee Ang, Jihyun Lee, Kwok Ng, Isabella dos Santos Alves, Sean Healy, Wendy Yajun Huang, Pauli Rintala, Jernice Sing Yee Tan, Yandan Wu, Hannah Yang, Eija Kärnä, Hyokju Maeng, André Lisandro Schliemann, Ding Ding

**Affiliations:** 1 School of Physical Education & Sports Science, South China Normal University, Guangzhou, China; 2 Adapted Physical Activity + Laboratory, South China Normal University, Guangzhou, China; 3 Department of Human Movement Sciences, Old Dominion University, Norfolk, United States; 4 Center for Movement, Health, & Disability, Old Dominion University, Norfolk, United States; 5 Department of Health and Physical Education, The Education University of Hong Kong, Hong Kong SAR, China; 6 Faculty of Physical Education, University of Campinas, São Paulo, Brazil; 7 Sport Singapore, Singapore, Singapore; 8 Department of Kinesiology, San José State University, San Jose, United States; 9 Physical Activity for Health Research Cluster, Department of Physical Education and Sport Sciences, University of Limerick, Limerick, Ireland; 10 Faculty of Education, University of Turku, Rauma, Finland; 11 School of Educational Sciences and Psychology, University of Eastern Finland, Joensuu, Finland; 12 School of Nursing, Psychotherapy and Community Health, Dublin City University, Dublin, Ireland; 13 Department of Sport, Physical Education and Health, Hong Kong Baptist University, Hong Kong SAR, China; 14 Faculty of Sport and Health Sciences, University of Jyväskylä, Jyväskylä, Finland; 15 School of Sports, Health and Leisure, Republic Polytechnic, Singapore, Singapore; 16 School of Physical Education & Sports Science, Fujian Normal University, Fuzhou, China; 17 Department of Adapted Physical Education, Baekseok University, Cheonan-si, South Korea; 18 Department of Kinesiology and Health, Georgia State University, Atlanta, United States; 19 Sydney School of Public Health, Faculty of Medicine and Health, The University of Sydney, New South Wales, Australia

**Keywords:** Movement behavior, Adolescent, Disability, Physical activity, Screen time, Sleep

## Abstract

**Background:**

Meeting daily guidelines for physical activity, screen time, and sleep duration is associated with a host of health indicators for youth. In this cross-sectional observational study, we investigated the associations between adherence to the movement guidelines and health-related outcomes among youth with autism spectrum disorder (ASD).

**Methods:**

Parents of youth with ASD (10–17 years) from seven countries and regions were invited to provide online proxy-reports for child’s movement behaviors (i.e., physical activity, sleep and screen time), and health-related outcomes (i.e., body mass index [BMI], general health, and quality of life). A series of multiple linear regression analyses were used to examine the associations between meeting movement guidelines and health-related outcomes, adjusted for covariates.

**Results:**

The final sample consisted of 1165 youth with ASD. Compared with youth meeting all three guidelines, a higher BMI *z*-score was observed in those who met no guidelines (B = 0.62, *P* = 0.04), “sedentary time only” (B = 0.60, *P* = 0.047), and “physical activity plus sleep only” (B = 0.85, *P* = 0.04). Compared with meeting all three guidelines, meeting no guidelines was associated with poorer general health (B = − 0.46, *P* = 0.02). Further, compared with youth meeting all three guidelines, a lower quality of life score was observed in those who met no guidelines (B = − 0.47, *P* = 0.02) and “physical activity only” (B = − 0.62, *P* = 0.03). Lastly, there were dose–response associations between the number of guidelines met and all three health-related outcomes (all *P*_trend_ < 0.05).

**Conclusions:**

In conclusion, meeting more 24-h movement guidelines was generally associated with more favorable health-related outcomes in youth with ASD. The low level of adherence to all three guidelines (2.0%) suggests the urgent need to promote the adoption of all the guidelines in this group.

## Background

Physical activity, sedentary behavior, and sleep are important modifiable behaviors that can impact the physical and psychological health of youth [1–4]. Traditionally studied in isolation, the importance of considering these three movement behaviors has been increasingly recognized by public health communities [4, 5]. The integrated 24-h movement paradigm recognizes that physical activity, sedentary behavior, and sleep are codependent health-enhancing behaviors that fall on a movement to nonmovement continuum, and together account for a 24-h daily cycle [3, 4]. Reflecting the collective, integrative nature of these movement behaviors, Tremblay and colleagues [4] proposed a 24-h movement framework, which includes the following daily guidelines for children and youth aged 5–17 years: (a) ≥ 60 min of moderate-to-vigorous physical activity, (b) ≤ 120 min of recreational screen time, and (c) 9 to 11 h of sleep per night for those aged 5–13 years, or 8 to 10 h of sleep per night for those aged 14–17 years. Current research demonstrates that meeting all three movement guidelines can have multiplicative effects, above and beyond meeting none or one guideline in isolation, on physiological (e.g., body mass index [BMI], systolic and diastolic blood pressure) and psychological (e.g., well-being, depression) outcomes [2, 3, 6–9]. However, alarmingly small proportions of youth appear to be meeting each of these guidelines. For example, Roman-Vinas and colleagues [6] examined the adherence to the three 24-h movement guidelines among 6,128 children and youth aged 9–11 years from 12 countries and found that just 7.2% of the total sample met all three guidelines, whereas 19% met none.

While research examining the prevalence and benefits of meeting multiple 24-h movement guidelines among youth is emerging [4, 5], the extension of this integrated framework to study the health behaviors among youth with disabilities, such as those with autism spectrum disorder (ASD), is limited. To date, studies have demonstrated that youth with ASD tend to engage in inadequate amounts of physical activity [10–13] and sleep [14, 15], and an overabundance of recreational screen time [16, 17], which may lead to undesirable physical and psychological outcomes, such as overweight or obesity [11, 18, 19], health concerns [19, 20], and poor quality of life [20, 21]. However, these studies have largely explored movement behaviors in isolation, and few have explored the prevalence of meeting multiple 24-h movement guidelines among youth with ASD. To the knowledge of the authors, only two studies, each contextualized in the US, have examined proportions of youth with ASD meeting the 24-h movement guidelines [22, 23]. For instance, Healy and colleagues [22] utilized data from 1008 youth with ASD from the 2016 National Survey of Children’s Health (NSCH) to examine adherence to the 24-h movement guidelines, and identified that 6.5% children (ages 6–12 years) and 4.4% adolescents (ages 13–17 years) met all guidelines, whereas about 12% of both groups met none. In a second analysis of the 2016 NSCH in the US, just 2.6% of 746 youth with ASD aged 10–17 years were identified as meeting all three guidelines concurrently, whereas 19.9% met no guidelines [23].

Although empirical data supports the integrative benefits of meeting the 24-h movement guidelines for physiological and psychological health indices among youth without disabilities [4, 5], support for the extension of this framework to at-risk groups, such as youth with ASD, is warranted. Examining the associations between meeting the 24-h movement guidelines and health indices can help inform prioritization and resource allocation, and guide health promotion in general, and specifically for youth with ASD. Taken together, in this cross-sectional study, we aimed first to examine the prevalence of meeting individual and combined guidelines for physical activity, screen time, and sleep duration among youth with ASD in seven countries and regions. Secondly, we aimed to examine associations between meeting none, one, two, or three of the 24-h movement guidelines and three health-related outcomes (i.e., BMI, general health, and quality of life) among youth with ASD.

## Methods

### Study design

This cross-sectional survey was conducted across seven countries and regions: Brazil, Finland, Hong Kong, Mainland China, Singapore, South Korea, and the US. Ethics approval was obtained from the Human Research Ethics Committee of the Education University of Hong Kong, Hong Kong SAR (Ref. no. A2019-2020–0431). For participating universities or institutions in other countries/regions, local research ethics guidelines were followed if required.

### Sampling and procedures

A standardized study protocol was followed. Convenience samples from each identified country/region were recruited through ASD associations, direct contact with special school principals, and social media. Participants were parents or guardians of youth with ASD. To be eligible for the present study, parents had to have a child with ASD who met the following criteria: (a) had doctor-diagnosed ASD; (b) aged 10 to 17 years; and (c) lived in one of the seven countries or regions.

Parents who were interested in participation were provided a URL link to an online survey package. In total, 1580 parents entered the online survey after reading an information sheet and providing electronic informed consent. Of those, 1210 (77%) continued responding to survey questions, whereas 370 (23%) exited the survey after completing informed consent without answering questions, submitting blank forms. All data were collected between 18 August 2020 and 30 April 2021.

### Measures and variables

All the survey items, if not otherwise specified, were adopted from the 2018 NSCH [24]. These NSCH items had been used previously in studies examining health behaviors via parent proxy-report of children with ASD [22, 23]. The survey form was initially prepared in English language and was translated into different languages following the standardized translation procedure [25]. The procedure involved an iterative process of translation and back-translation. For quality control, a standardized translation protocol was prepared and followed by the research team.

#### Demographics

The diagnosis of ASD was measured using one question, “Has a doctor or other health care provider ever told you that your child has Autism or Autism Spectrum Disorder?”. Parents rated this question on a binary scale (yes, no). If the answer was “yes”, parents were required to rate the severity of ASD (i.e., mild, moderate, or severe). Parents also reported their child’s age, sex, height, and weight.

#### Outcomes

Three variables (i.e., BMI, general health, and quality of life) were identified as outcome variables for this study. Proxy-report of child’s height and weight information was converted to age- and gender-specific BMI *z*-scores (*z*BMI) following the World Health Organization (WHO) growth standards [26]. *z*BMI was further categorized into underweight (< 5th percentile), healthy weight (5th to 84th percentile), overweight (85th to 94th percentile), and obesity (≥ 95th percentile).

General health was measured using one question, “In general, how would you describe this child’s health?”. Parents rated this question on a 5-point Likert scale (1 = “excellent”, 5 = “poor”) with responses reversely coded, so that higher scores represented better general health. Quality of life was assessed using one question, “Is this child satisfied with his/her life?”, which was adopted from previous research [27]. Parents rated this question on a 5-point Likert scale (1 = “not at all”, 5 = “totally”), and a higher score represented a greater level of quality of life.

#### Predictors

Physical activity was measured using the question, “During the past week, on how many days did this child exercise, play a sport, or participate in physical activity for at least 60 min?”. Response options were: “0 days”, “1 day”, “2 days”, “3 days”, “4 days”, “5 days”, “6 days”, and “everyday”. Sleep was assessed using the question, “During the past week, how many hours of sleep did this child get on an average weeknight?”. Response options were: “less than 6 h”, “6 h”, “7 h”, “8 h”, “9 h”, “10 h”, “11 h”, and “12 or more hours”. Screen time was measured using the question, “On average, how many total hours per day did your child watch TV, use the computer, use social media and inactive play video games, during their free time over the last week?” [28]. The latter question had been used by parents to report their children’s screen time in a national survey [28]. Response options were: “none”, “less than 1 h”, “1 h”, “2 h”, “3 h”, and “4 or more hours”.

Responses to each movement behavior variable was dichotomized into “meeting the guideline” *vs*. “not meeting the guideline” [4, 29, 30]. Specifically, meeting the guideline refers to reporting physical activity for at least 60 min “everyday”, screen time for “2 h per day or less” [4, 29], and sleep for “8 to 10 h” for 14–17 years and “9 h to 11 h” for 10–13 years [4, 30].

## Statistical analyses

Statistical analyses were performed with SPSS (Version 25, IBM; Armonk, New York). First, we removed incomplete observations and continuous outliers (*z* > 3.29 or *z* < − 3.29). Second, descriptive statistics, means (*M*) and standard deviation (*SD*), or frequencies and percentages, were calculated for each continuous and categorical variable. Third, a series of multiple linear regression analyses were conducted to determine the associations between (a) number of 24-h movement guidelines met and (b) specific combinations of guidelines met with each health-related outcome, adjusted for age, sex, physician-rated severity of ASD, and country/region [31–34]. Meeting all three guidelines was used as the reference group. Lastly, several trend analyses were conducted to determine whether meeting more guidelines was associated with more favorable outcomes. All regression models were adjusted for country, age, gender, and severity level of ASD. Statistical significance was set at P < 0.05.

## Results

### Descriptive data

Initially, a total of 1210 youth with ASD from seven countries or regions met the inclusion criteria and submitted survey responses. After removing incomplete observations and outliers, a final sample of 1165 youth with ASD were included for analysis. Table 1 presents participant demographics and descriptive statistics. The sample had a mean age of 13.1 years (*SD* = 2.2), with approximately one-quarter being female (24.4%) and around half having mild ASD (48.8%).

**Table 1:** Demographic characteristics and descriptive statistics

Characteristics	Overall^b^ (*n* = 1,165)	Brazil (*n* = 228)	Finland (*n* = 278)	Hong Kong (*n* = 96)	Mainland China (*n* = 186)	Singapore (*n* = 89)	South Korea (*n* = 202)	US (*n* = 86)
Age, year (10–17)^a^	13.08 (2.18)	13.01 (2.09)	13.34 (2.06)	13.50 (2.50)	12.43 (2.15)	13.20 (2.62)	13.01 (2.06)	13.34 (2.01)
Gender								
Male	881 (75.6%)	199 (87.3%)	185 (66.5%)	85 (88.5%)	153 (82.3%)	71 (79.8%)	125 (61.9%)	63 (73.3%)
Female	284 (24.4%)	29 (12.7%)	93 (33.5%)	11 (11.5%)	33 (17.7%)	18 (20.2%)	77 (38.1%)	23 (26.7%)
Severity level of ASD								
Mild	568 (48.8%)	132 (57.9%)	140 (50.4%)	67 (69.8%)	54 (29.0%)	46 (51.7%)	92 (45.5%)	37 (43.0%)
Moderate or severe	597 (51.2%)	96 (42.1%)	138 (49.6%)	29 (30.2%)	132 (71.0%)	43 (48.3%)	110 (54.5%)	49 (57.0%)
Physical activity guideline								
Yes	84 (7.2%)	9 (3.9%)	26 (9.4%)	6 (6.3%)	28 (15.1%)	7 (7.9%)	3 (1.5%)	5 (5.8%)
Screen time guideline								
Yes	540 (46.4%)	46 (20.2%)	77 (27.7%)	43 (44.8%)	143 (76.9%)	39 (43.8%)	164 (81.2%)	28 (32.6%)
Sleep guideline								
Yes	651 (55.9%)	139 (61.0%)	189 (68.0%)	48 (50%)	84 (45.2%)	39 (43.8%)	104 (51.5%)	48 (55.8%)
Number of guidelines meet								
None	251 (21.5%)	64 (28.1%)	63 (22.7%)	26 (27.1%)	22 (11.8%)	30 (33.7%)	21 (10.4%)	25 (29.1%)
One of three	576 (49.4%)	137 (60.1%)	146 (52.5%)	43 (44.8%)	83 (44.6%)	35 (39.3%)	91 (45.0%)	41 (47.7%)
Two of three	315 (27.0%)	24 (10.5%)	61 (21.9%)	27 (28.1%)	71 (38.2%)	22 (24.7%)	90 (44.6%)	20 (23.3%)
All three	23 (2.0%)	3 (1.3%)	8 (2.9%)	0 (0%)	10 (5.4%)	2 (2.2%)	0 (0%)	0 (0%)
BMI z-score	0.60 (1.38)	0.90 (1.35)	0.50 (1.42)	0.56 (1.26)	0.53 (1.48)	0.24 (1.59)	0.60 (1.06)	0.71 (1.53)
Body weight status								
Underweight	76 (6.5%)	9 (3.9%)	20 (7.2%)	4 (4.2%)	17 (9.1%)	13 (14.6%)	3 (1.5%)	10 (11.6%)
Normal weight	611 (52.4%)	97 (42.5%)	152 (54.7%)	50 (52.1%)	88 (47.3%)	47 (52.8%)	142 (70.3%)	35 (40.7%)
Overweight	194 (16.7%)	50 (21.9%)	37 (13.3%)	21 (21.9%)	38 (20.4%)	11 (12.4%)	24 (11.9%)	13 (15.1%)
Obesity	284 (24.4%)	72 (31.6%)	69 (24.8%)	21 (21.9%)	43 (23.1%)	18 (20.2%)	33 (16.3%)	28 (32.6%)
General health (1–5)^a^	3.27 (0.97)	3.66 (0.93)	3.28 (0.94)	2.92 (0.88)	2.56 (0.89)	3.26 (0.95)	3.51 (0.77)	3.57 (0.91)
Quality of life (1–5)^a^	3.33 (0.93)	3.50 (1.00)	3.29 (0.94)	3.43 (0.95)	3.22 (0.92)	3.54 (0.88)	3.03 (0.78)	3.59 (0.83)

ASD: autism spectrum disorder; BMI: body mass index

^a^Possible range

^b^Values are mean (*SD)* or *n* (%)

According to the 24-h movement guidelines, only a small proportion of the sample (7.2%, range = 1.5% [South Korea] to 15.1% [Mainland China]) met the physical activity guideline. On average, around half of the sample met the screen time guideline (46.4%, range = 20.2% [Brazil] to 81.2% [South Korea]) and the sleep guideline (55.9%, range = 43.8% [Singapore] to 68.0% [Finland]). The largest proportion of youth met only one guideline (49.4%, range = 39.3% [Singapore] to 60.1% [Brazil]), followed by meeting two (27%, range = 10.5% [Brazil] to 44.6% [South Korea]) and zero guidelines (21.5%, range = 10.4% [South Korea] to 33.7% [Singapore]). Only 2% of the sample met all three guidelines. Particularly, none of the youth with ASD from Hong Kong, South Korea, and the US met all three guidelines. Regarding body weight status, 16.7% of the sample were overweight (range = 11.9% [South Korea] to 21.9% [Hong Kong]) and 24.4% were obese (range = 16.3% [South Korea] to 32.6% [US]). We found a moderate level of general health (*M* = 3.27, *SD* = 0.97, range = 2.92 [Hong Kong] to 3.66 [Brazil]) and quality of life (*M* = 3.33, *SD* = 0.93, range = 3.03 [South Korea] to 3.59 [US]).

### Associations between meeting the guidelines and outcomes

Table 2 presents the associations between meeting the 24-h movement guidelines and health-related outcomes. Compared to youth meeting all three guidelines, those who met no guidelines had significantly higher *z*BMI (B = 0.63, 95% CI [0.05, 1.22], *P* = 0.04) as well as lower general health (B = − 0.45, 95% CI [− 0.83, − 0.06], *P* = 0.02) and quality of life (B = − 0.46, 95% CI [− 0.85, − 0.07], *P* = 0.02).

**Table 2:** Associations between meeting movement guidelines and health-related outcomes

	BMI *z*-score		General health		Quality of life	
	B (95%CI)	*P*	B (95%CI)	*P*	B (95%CI)	*P*
Number of guidelines (*n*)						
None (251)	**0.63 (0.05,1.22)**	**0.04**	**− 0.45 (− 0.83, − 0.06)**	**0.02**	**− 0.46 (− 0.85,− 0.07)**	**0.02**
One of three (576)	0.55 (− 0.03, 1.12)	0.06	− 0.24 (− 0.61, 0.14)	0.22	− 0.36 (− 0.74, 0.02)	0.06
Two of three (315)	0.46 (− 0.12, 1.04)	0.12	− 0.16 (− 0.54, 0.22)	0.41	− 0.15 (− 0.53, 0.24)	0.46
All three (23)	Reference		Reference		Reference	
Trend analysis	**− 0.11 (− 0.22, − 0.004)**	**0.04**	**0.14 (0.07, 0.21)**	** < 0.001**	**0.16 (0.09, 0.23)**	** < 0.001**
*R*^*2*^	3.9%		16.8%		6.3%	
Specific combinations of guidelines (*n*)						
None (251)	**0.62 (0.03, 1.21)**	**0.04**	**− 0.46 (− 0.84, − 0.08)**	**0.02**	**− 0.47 (− 0.86, − 0.08)**	**0.02**
Physical activity only (17)	0.18 (− 0.68, 1.04)	0.68	0.29 (− 0.27, 0.85)	0.30	**− 0.62 (− 1.19, − 0.05)**	**0.03**
Sleep only (336)	0.51 (− 0.07, 1.10)	0.09	− 0.31 (− 0.69, 0.07)	0.11	− 0.38 (− 0.77, 0.01)	0.054
Sedentary time only (223)	**0.60 (0.01, 1.19)**	**0.047**	− 0.19 (− 0.57, 0.20)	0.34	− 0.33 (− 0.73, 0.06)	0.10
Physical activity + sleep only (21)	**0.85 (0.05, 1.66)**	**0.04**	0.29 (− 0.24, 0.82)	0.28	− 0.10 (− 0.64, 0.44)	0.72
Physical activity + sedentary time only (23)	0.70 (− 0.09, 1.49)	0.08	0.11 (− 0.40, 0.63)	0.66	0.05 (− 0.48, 0.57)	0.87
Sleep + sedentary time only (271)	0.40 (− 0.18, 0.99)	0.18	− 0.22 (− 0.61, 0.16)	0.25	− 0.17 (− 0.56, 0.22)	0.39
All three (23)	Reference		Reference		Reference	
*R*^*2*^	4.3%		18.1%		6.5%	

ASD: autism spectrum disorder; BMI: body mass index; B: unstandardized coefficients; CI: confidence interval

All models are adjusted for country, age, gender, and severity level of ASD. Statistically significant associations (*P* < 0.05) are highlighted in bold

Regarding the associations between specific combinations of guidelines met and outcomes, meeting “none” (B = 0.62, 95% CI [0.03, 1.21], *P* = 0.04), “sedentary time only” (B = 0.60, 95% CI [0.01, 1.19], *P* = 0.047) and “physical activity plus sleep only” (B = 0.85, 95% CI [0.05, 1.66], *P* = 0.04) were significantly associated with higher *z*BMI than meeting all three guidelines. None of the associations between specific combinations of guidelines met and general health were statistically significant except for the comparison between “none” and “all three” (B = − 0.46, 95% CI [− 0.84, − 0.08], *P* = 0.02). Meeting no guidelines (B = − 0.47, 95% CI [− 0.86, − 0.08], *P* = 0.02) and “physical activity only” (B = − 0.62, 95% CI [− 1.19, − 0.05], *P* = 0.03) were significantly associated with a lower quality of life level as compared to meeting all three guidelines.

Finally, the results of the trend analysis suggested a significant dose–response association between the number of guidelines met and (a) *z*BMI (B = − 0.11, 95% CI [− 0.22, − 0.004], *P*_trend_ = 0.04, *R*^*2*^ = 3.9%), (b) quality of life (B = 0.16, 95% CI [0.09, 0.23], *P*_trend_ < 0.001, *R*^*2*^ = 6.3%), and (c) general health (B = 0.14, 95% CI [0.07, 0.21], *P*_trend_ < 0.001, *R*^*2*^ = 16.8%).

## Discussion

A focus on the integrative nature of the 24-h movement behaviors has represented a paradigm shift which reflects the concept that “the whole day matters” [4] for health promotion. This contemporary framework is supported by an emerging body of literature suggesting that meeting physical activity, screen time, and sleep duration guidelines can have synergistic benefits for youth [2, 3, 6–9]. The current study extends this contemporary framework to youth with ASD across seven countries or regions. Overall, only 2% of the sample (range = 0% to 5.4%) in this study met all three 24-h movement guidelines. This figure is substantially smaller than adherence estimates among youth without ASD, such as the 7.2% reported by Roman-Vinas and colleagues in their international sample [6], and it is consistent with low adherence rates reported for youth with ASD in the US [23]. This trend appears consistent across countries or regions included in this analysis, with less than 3% of samples from six of the seven countries or regions meeting all three 24-h movement guidelines. Mainland China is the exception, in this instance, where 5.4% of participants met all three guidelines, a rate that is nearly double that of any other country or region included in this analysis. Interestingly, in this study participants from Mainland China are also among the highest adherers to both the physical activity and screen time guidelines, which perhaps results from specific cultural values, social norms, and educational systems [35]. On the other hand, some countries or regions (i.e., Hong Kong, South Korea, and the US) had zero participants meet one of the three guidelines, highlighting the significance of recruiting participants from different centers. Failure to meet all three 24-h movement guidelines, as is the case for approximately 98% of the total sample in the current study, may influence high rates of physical or mental health concerns among youth with ASD [2, 3, 6–9]. For example, high rates of overweight and obesity have been found among youth with ASD [18, 19], including 41% among the current sample. It should be noted that the usage of psychotropic medications was not considered in the present survey. These medications may have negative influences on body weight gain in youth with ASD [36]. Overall, these findings reinforce the need to develop, test, and implement intervention programs that seek to improve the patterns of physical activity, sedentary behavior, and sleep in youth with ASD.

The present analysis allows us to identify specific health behaviors most in need of intervention. As such, of particular concern for youth with ASD is the low level of adherence to the physical activity guideline. That is, while approximately half of the total sample met screen time (46.4%) and sleep duration (55.9%) guidelines, only 7.2% met the physical activity guideline. This figure is generally lower than those reported among children and youth without disabilities [3, 6, 9]. Across countries or regions, this figure ranged from just 1.5% in South Korea to 15.1% in Mainland China. This finding may not be surprising, though, as prior analyses situated in this paradigm have identified that meeting the physical activity guideline is the biggest concern for youth with disabilities [37, 38], including those with ASD [22]. In addition, a multitude of studies have identified that by adolescence, youth with ASD tend not to engage in regular physical activity, particularly when compared to peers without disabilities [10–12, 16, 39]. A plethora of factors have been identified that may contribute to low physical activity engagement among adolescents with ASD, including time and financial constraints, a lack of opportunities, and social barriers associated with the stress that arises from engaging in and initiating social interactions during activities [16, 40]. Taken together, the current study along with prior research [10–12, 22, 37, 39] make it clear that adherence to physical activity guidelines is problematic for youth with ASD. Therefore, physical activity engagement should be the focus of behavioral interventions to improve the health of this population.

A growing body of research has shown that while a minority of youth meet all three 24-h movement guidelines, meeting those behaviors is associated with favorable physiological and psychological health outcomes [2, 3, 7–9]. The current study is the first to extend this line of inquiry to youth with ASD. Consistent with prior research on youth without disabilities [7–9], as well as those with disabilities broadly defined [35], participants in this analysis who met all three 24-h movement guidelines had more favorable *z*BMI scores, general health scores, and quality of life scores than those meeting none of the guidelines. In addition, dose–response associations between the number of guidelines met and *z*BMI, general health, and quality of life were evident. These findings are important, as they suggest that adhering to the 24-h movement guidelines may help reduce prevalent psychological and physical health issues previously identified among youth with ASD [18–20].

This study had several strengths, including an international sample of youth with ASD from culturally and geographically diverse countries and regions and the number of health-related outcomes examined in this understudied group. However, several limitations need to be acknowledged. First, our study, based on the convenience sample, is subject to selection bias. Also, the difference in the number of participants among countries may partly reflect response bias. Therefore, one may not be able to infer our findings to the populations of youth with ASD aged 10 to 17. However, it is important to note that recruitment of participants with ASD could be challenging. A recent systematic review of correlates of physical activity and sedentary behavior found that the average sample size across included studies was 41 children and youth with ASD [41]. Second, a cross-sectional design does not allow us to make casual inferences on the associations between adherence to the 24-h movement guidelines and health-related outcomes. In fact, research centered on youth without disabilities supports assertions that overweight and obesity could have a bi-directional association with selected health behaviors (e.g., physical activity, screen time) [42]. However, longitudinal analyses examining these bi-directional associations among youth with ASD are unavailable. Future studies should consider prospective or experimental study design to better infer causality. Finally, parental proxy-reports are prone to response bias, particularly with socially desirable constructs such as physical activity or weight status [43]. However, it is worth noting that proxy-report has been commonly used in public health surveillance (e.g., NSCH). Future work should consider supplementing proxy reports with objective measures, such as accelerometers, to improve measurement validity and capture further information, such as the timing of movement behaviors and the intensity of physical activity.

## Conclusions

In conclusion, this study demonstrated dose–response relationships between the number of 24-h guidelines met and more favorable health-related outcomes in an international (7-country/region) sample of youth with ASD. The low adherence to all three guidelines, and in particular physical activity, and the strong association between guideline adherence and health-related outcomes, suggest the pressing need to promote the adoption of all the guidelines in youth with ASD.

## Data Availability

Data described in this manuscript, code book, and analytic code will be made available upon request pending approval of a data use agreement.

## Abbreviations

ASD: Autism spectrum disorder
BMI: Body mass index
NSCH: National Survey of Children’s Health
WHO: World Health Organization
B: Unstandardized coefficients
CI: Confidence interval
